# Optimization of Heavy Metals Biosorption *via* Artificial Neural Network: A Case Study of Cobalt (II) Sorption by *Pseudomonas alcaliphila* NEWG-2

**DOI:** 10.3389/fmicb.2022.893603

**Published:** 2022-05-31

**Authors:** Ashraf Elsayed, Zeiad Moussa, Salma Saleh Alrdahe, Maha Mohammed Alharbi, Abeer A. Ghoniem, Ayman Y. El-khateeb, WesamEldin I. A. Saber

**Affiliations:** ^1^Botany Department, Faculty of Science, Mansoura University, Mansoura, Egypt; ^2^Department of Microbiology, Soils, Water and Environment Research Institute, Agricultural Research Center, Giza, Egypt; ^3^Department of Biology, Faculty of Science, University of Tabuk, Tabuk, Saudi Arabia; ^4^Department of Agricultural Chemistry, Faculty of Agriculture, Mansoura University, Mansoura, Egypt

**Keywords:** cobalt, biosorption, *Pseudomonas alcaliphila* NEWG-2, definitive screening design, artificial neural network

## Abstract

The definitive screening design (DSD) and artificial neural network (ANN) were conducted for modeling the biosorption of Co(II) by *Pseudomonas alcaliphila* NEWG-2. Factors such as peptone, incubation time, pH, glycerol, glucose, K_2_HPO_4_, and initial cobalt had a significant effect on the biosorption process. MgSO_4_ was the only insignificant factor. The DSD model was invalid and could not forecast the prediction of Co(II) removal, owing to the significant lack-of-fit (*P* < 0.0001). Decisively, the prediction ability of ANN was accurate with a prominent response for training (R^2^ = 0.9779) and validation (R^2^ = 0.9773) and lower errors. Applying the optimal levels of the tested variables obtained by the ANN model led to 96.32 ± 2.1% of cobalt bioremoval. During the biosorption process, Fourier transform infrared spectroscopy (FTIR), energy-dispersive X-ray spectroscopy, and scanning electron microscopy confirmed the sorption of Co(II) ions by *P. alcaliphila*. FTIR indicated the appearance of a new stretching vibration band formed with Co(II) ions at wavenumbers of 562, 530, and 531 cm^–1^. The symmetric amino (NH_2_) binding was also formed due to Co(II) sorption. Interestingly, throughout the revision of publications so far, no attempt has been conducted to optimize the biosorption of Co(II) by *P. alcaliphila via* DSD or ANN paradigm.

## Introduction

The potentiality of industrial effluents containing heavy metal ions, such as copper, chromium, lead, and nickel, toward the environment had been reported to be a cause of the virulent impact on a variety of living organisms (i.e., plants, animals, and human beings) (Alotaibi et al., 2021). These metals can accumulate in tissues of animals, and also in the human body (Alfadaly et al., 2021), causing disruption of cell membranes, lipid peroxidation, inhibition of oxidative phosphorylation, protein denaturation, and alteration of nucleic acids structures (Xie et al., 2016).

Cobalt is one of these metals with consideration as a carcinogenic agent, causing several types of cancer, including lung cancer (Viegas et al., 2022). The emission of cobalt and other metals into the environment could be associated with some industries, e.g., alloy production, electroplating generations of gas turbines, petrochemical industries, metal plating activities, and mining processes (Soualili et al., 2008). Moreover, cobalt could be produced from power-generating nuclear reactors, e.g., pressurized light water reactors and pressurized heavy water reactors. Co(II) is a major contributor to health threats because it has a long half-life time (5.27 years) and high-energy (1.17 and 1.33 MeV) (Raghu et al., 2008). Furthermore, the clinical symptoms of cobalt-associated diseases include a neurotoxicological disorder, carcinogenicity of the thyroid gland, erythropoietic, and genotoxicity in human beings (Lison, 2022). Cobalt was also found to be a cause of many serious diseases, such as, nausea, vomiting, bone defects, blood pressure disorder, and heart defects (Salmani et al., 2020). Additionally, the toxicity of cobalt could be extended to plants causing a reduction in shoot and root growth, chlorophyll content, uptake of minerals, and antioxidant enzymes (Mahey et al., 2020; Banerjee and Bhattacharya, 2021). Consequently, the industrial effluents should not be exceeding 1.0 and/or 0.05 mg/L of Co(II) (Saad et al., 2020). Wherein, the rules and regulations must be set up for the discharge of contaminants to be a safeguard for the ecosystem.

Currently, the technical procedures of effluents remediation are following the biosorption approach, which could be one of the propitious technologies; these strategies are relatively simple and inexpensive with an efficiency of sequestering the toxicity of metals. The other physicochemical technologies are either ineffective or use costly chemicals (Dharanguttikar, 2018; Ghoniem et al., 2020; Rashid et al., 2021). Overall, microorganisms are effectively bioabsorptive and biodegrade heavy metals and other pollutants (Sonune, 2021), further, they are economic and eco-friendly. Therefore, numerous investigating studies suggested a lot of bacteria, fungi, and seaweeds for the sorption of metals and other environmental contaminants (Meez and Kyzas, 2021).

The potentiality of the biosorption process involves the accumulation of metal ions into the cell wall and chases into the cell (Brar et al., 2006; Ozdemir et al., 2020). Additionally, the management of the genetic and biochemical capacity of microorganisms for the bioremediation process of heavy metals has been investigated by Valls and De Lorenzo (2002), who defined the advantages of biological procedures, i.e., specify suitability, and potentiality for the genetic upgrade. The pertinence of living organisms and biopolymers has been involved in a model of biosorption, where the biosorbents could be viewed as natural ion-exchange agents that contain weakly acidic and basic groups (Gutnick and Bach, 2000). For example, species of seaweeds and marine bacteria were found to accumulate and uptake relatively high metal concentrations (Iyer et al., 2005; Hu et al., 2010; Jarvis and Bielmyer-Fraser, 2015). Khraisheh et al. (2020) investigated the efficiency of *Pseudomonas putida* in the remediation of cobalt from contaminated effluents, by which some bacterial components, e.g., exopolysaccharides, associated with *Enterobacter cloaceae* acted as a chelating agent for cobalt, copper, and cadmium (Iyer et al., 2005).

Otherwise, to successfully achieve the optimized leaching of heavy metals, the multivariate statistical optimizations were adopted by developing an empirical experiment based on the optimization model, where the interaction between dependent and independent variables was identified (Kamalini et al., 2018). The statistical procedures that could be preferred in the optimization process for heavy metals management are response surface methodology and the artificial intelligence-based black-box model [artificial neural network (ANN)] (Shanmugaprakash et al., 2018). The ANN approach was found to be widely exhausted in the optimization of fermentation processes (Dziuba and Nalepa, 2012) and was one of profoundly statistical analyses with output dissimilar to the response surface methodology. The prettiness of ANNs as empirical modeling is owing to their capability to extract, accurate, and regardless of the degree of nonlinearity breathing between independent and dependent variables through the training of network as modeling for predictive the optimized response value (Poirazi et al., 2007; Nor et al., 2017).

Additionally, the ANN has emerged as an alternative tool for the nonlinear multivariate modeling (Desai et al., 2005). The aptitude of ANN is generic in structure and acquires the skill to gain knowledge from historical data. Moreover, the chief recompenses of ANN compared with other experimental designs are (i) ANN does not require a previous description of the suitable fitting function and (ii) ANN has entire estimate capability, i.e., it can guess almost all sorts of nonlinear functions, including quadratic one; it could be of thought that the ANN requires a much greater number of experimental trials to assemble an efficient model. ANN can also perform thoroughly even with fairly fewer data. A few case studies were investigated by response surface methodology and ANN using the same experimental design, where the ANN models have regularly functioned better than response surface methodology (Baş and Boyaci, 2007; Saber et al., 2021). Generally, the ANNs have been employed successfully in a variety of biotechnological processes (Bingöl et al., 2012; Ghritlahre and Prasad, 2018).

Definitive screening design (DSD) is a new statics procedure that has been introduced lately in biological processing. It could effectually estimate the main effects that are impartial to any quadratic effects and two-factor interaction (Lin, 2015; Tai et al., 2015). Therefore, the DSD could be afforded to go along with experiments that seemed to be unnecessary in a lot of circumstances, with avoiding the confusion of effects. It can characterize factors having a nonlinear or curvilinear effect on the response (Jones and Nachtsheim, 2011; Lin, 2015).

To the best of our knowledge, there are relatively few reported cases that conquer the optimized biosorption process of cobalt, especially by *Pseudomonas* spp. Therefore, our investigated study has been designed to optimize the removal of Co(II) by *Pseudomonas alcaliphila* NEWG-2 using the DSD and ANN, with a determination of the biosorption process of Co(II) into the bacterial cell.

## Materials and Methods

### Bacterium and Biosorption Medium

*Pseudomonas alcaliphila* NEWG-2 strain, previously identified using (DDBJ/EMBL-Bank/Gen Bank database under the accession number of MN025267) (El-Naggar et al., 2020), was used throughout this investigation.

The biosorption medium of Atlas and Snyder (2006) was used with some modifications. Unless otherwise stated, the medium components were used at the central level as reported in Table 1 without cobalt (CoSO_4_⋅7H_2_O, Aldrich) and then sterilized at 121°C for 15 min. Glucose was sterilized separately by a membrane filter (0.22 μm) and was added to the medium after sterilization. For preservation, the bacterial strain was cultured on slants of the same medium supported with 15 g agar and incubated at 28 ± 1°C for 48 h. The bacteria were sub-cultured periodically and conserved at 4°C.

**TABLE 1:** The definitive screening design (DSD) matrix of the independent factors, and the experimental data of Co (II) bioremoval by *P. alcaliphila* NEWG-2 as well as the corresponding predicted and residual values obtained from DSD and artificial neural network (ANN) models.

Run		Coded level of the independent variable in design matrix								Response of cobalt removal, %				
										Actual	DSD		ANN	
				
		X1	X2	X3	X4	X5	X6	X7	X8		Predicted	Residual	Predicted	Residual
1	Validation	0	1	1	1	1	1	1	1	69.49	71.78	−2.30	69.65	−0.16
2	Training	0	−1	−1	−1	−1	−1	−1	−1	71.88	70.65	1.23	72.31	−0.43
3	Training	1	0	−1	−1	−1	−1	1	1	77.75	79.46	−1.71	77.72	0.03
4	Training	−1	0	1	1	1	1	−1	−1	64.79	62.98	1.81	64.43	0.36
5	Training	1	−1	0	−1	1	1	−1	−1	59.90	62.10	−2.20	59.59	0.31
6	Validation	−1	1	0	1	−1	−1	1	1	81.66	80.34	1.32	83.34	−1.68
7	Validation	1	−1	−1	0	1	1	1	1	65.66	63.38	2.28	66.43	−0.77
8	Training	−1	1	1	0	−1	−1	−1	−1	77.82	79.06	−1.24	78.45	−0.63
9	Training	1	−1	1	1	0	−1	−1	1	82.57	81.28	1.29	82.24	0.33
10	Training	−1	1	−1	−1	0	1	1	−1	58.72	61.16	−2.43	59.58	−0.85
11	Training	1	−1	1	1	−1	0	1	−1	62.23	65.54	−3.31	61.64	0.59
12	Validation	−1	1	−1	−1	1	0	−1	1	77.31	76.89	0.42	76.72	0.59
13	Validation	1	1	−1	1	−1	1	0	−1	74.89	72.65	2.24	74.74	0.15
14	Training	−1	−1	1	−1	1	−1	0	1	68.31	69.79	−1.48	69.10	−0.79
15	Validation	1	1	−1	1	1	−1	−1	0	96.69	100.28	−3.58	97.05	−0.35
16	Validation	−1	−1	1	−1	−1	1	1	0	42.30	42.16	0.14	42.52	−0.23
17	Validation	1	1	1	−1	−1	1	−1	1	66.38	66.85	−0.47	66.49	−0.11
18	Validation	−1	−1	−1	1	1	−1	1	−1	75.22	75.59	−0.36	75.56	−0.34
19	Training	1	1	1	−1	1	−1	1	−1	93.91	90.64	3.27	93.25	0.66
20	Validation	−1	−1	−1	1	−1	1	−1	1	51.43	51.79	−0.36	50.03	1.40
21	Training	0	0	0	0	0	0	0	0	71.69	71.22	0.47	72.51	−0.83
22	Validation	0	0	0	0	0	0	0	0	73.90	71.22	2.68	72.51	1.39
23	Training	0	0	0	0	0	0	0	0	72.11	71.22	0.89	72.51	−0.40
24	Validation	0	0	0	0	0	0	0	0	73.32	71.22	2.11	72.51	0.81
25	Training	0	0	0	0	0	0	0	0	70.54	71.22	−0.68	72.51	−1.98
26	Training	0	1	1	1	1	1	1	1	68.49	71.78	−3.30	69.65	−1.16
27	Validation	0	−1	−1	−1	−1	−1	−1	−1	70.87	70.65	0.22	72.31	−1.44
28	Training	1	0	−1	−1	−1	−1	1	1	75.74	79.46	−3.72	77.72	−1.98
29	Training	−1	0	1	1	1	1	−1	−1	63.79	62.98	0.81	64.43	−0.64
30	Validation	1	−1	0	−1	1	1	−1	−1	57.89	62.10	−4.21	59.59	−1.70
31	Training	−1	1	0	1	−1	−1	1	1	84.66	80.34	4.32	83.34	1.32
32	Validation	1	−1	−1	0	1	1	1	1	64.66	63.38	1.28	66.43	−1.78
33	Training	−1	1	1	0	−1	−1	−1	−1	79.82	79.06	0.76	78.45	1.37
34	Training	1	−1	1	1	0	−1	−1	1	80.50	81.28	−0.78	82.24	−1.74
35	Training	−1	1	−1	−1	0	1	1	−1	57.72	61.16	−3.44	59.58	−1.86
36	Training	1	−1	1	1	−1	0	1	−1	60.24	65.54	−5.31	61.64	−1.41
37	Validation	−1	1	−1	−1	1	0	−1	1	75.31	76.89	−1.59	76.72	−1.41
38	Training	1	1	−1	1	−1	1	0	−1	73.89	72.65	1.24	74.74	−0.86
39	Validation	−1	−1	1	−1	1	−1	0	1	66.30	69.79	−3.49	69.10	−2.80
40	Training	1	1	−1	1	1	−1	−1	0	94.69	100.28	−5.59	97.05	−2.36
41	Training	−1	−1	1	−1	−1	1	1	0	44.30	42.16	2.14	42.52	1.78
42	Training	1	1	1	−1	−1	1	−1	1	64.38	66.85	−2.47	66.49	−2.11
43	Training	−1	−1	−1	1	1	−1	1	−1	74.22	75.59	−1.37	75.56	−1.35
44	Training	1	1	1	−1	1	−1	1	−1	90.90	90.64	0.26	93.25	−2.35
45	Training	−1	−1	−1	1	−1	1	−1	1	49.43	51.79	−2.36	50.03	−0.60
46	Validation	0	0	0	0	0	0	0	0	69.68	71.22	−1.54	72.51	−2.83
47	Training	0	0	0	0	0	0	0	0	75.91	71.22	4.69	72.51	3.40
48	Training	0	0	0	0	0	0	0	0	70.11	71.22	−1.11	72.51	−2.40
49	Training	0	0	0	0	0	0	0	0	72.32	71.22	1.10	72.51	−0.19
50	Validation	0	0	0	0	0	0	0	0	69.53	71.22	−1.69	72.51	−2.98
51	Training	0	1	1	1	1	1	1	1	70.49	71.78	−1.29	69.65	0.84
52	Training	0	−1	−1	−1	−1	−1	−1	−1	72.89	70.65	2.24	72.31	0.58
53	Training	1	0	−1	−1	−1	−1	1	1	79.75	79.46	0.29	77.72	2.03
54	Validation	−1	0	1	1	1	1	−1	−1	65.79	62.98	2.81	64.43	1.36
55	Training	1	−1	0	−1	1	1	−1	−1	61.90	62.10	−0.20	59.59	2.31
56	Training	−1	1	0	1	−1	−1	1	1	78.66	80.34	−1.68	83.34	−4.68
57	Training	1	−1	−1	0	1	1	1	1	66.66	63.38	3.28	66.43	0.23
58	Training	−1	1	1	0	−1	−1	−1	−1	75.82	79.06	−3.24	78.45	−2.63
59	Training	1	−1	1	1	0	−1	−1	1	84.64	81.28	3.36	82.24	2.40
60	Validation	−1	1	−1	−1	0	1	1	−1	59.73	61.16	−1.43	59.58	0.15
61	Training	1	−1	1	1	−1	0	1	−1	64.24	65.54	−1.30	61.64	2.60
62	Training	−1	1	−1	−1	1	0	−1	1	79.31	76.89	2.42	76.72	2.59
63	Training	1	1	−1	1	−1	1	0	−1	75.90	72.65	3.25	74.74	1.16
64	Training	−1	−1	1	−1	1	−1	0	1	70.32	69.79	0.53	69.10	1.22
65	Training	1	1	−1	1	1	−1	−1	0	98.69	100.28	−1.59	97.05	1.64
66	Training	−1	−1	1	−1	−1	1	1	0	40.30	42.16	−1.86	42.52	−2.22
67	Training	1	1	1	−1	−1	1	−1	1	68.39	66.85	1.54	66.49	1.90
68	Training	−1	−1	−1	1	1	−1	1	−1	76.23	75.59	0.64	75.56	0.67
69	Training	1	1	1	−1	1	−1	1	−1	96.92	90.64	6.28	93.25	3.67
70	Validation	−1	−1	−1	1	−1	1	−1	1	53.44	51.79	1.65	50.03	3.41
71	Training	0	0	0	0	0	0	0	0	73.70	71.22	2.48	72.51	1.19
72	Validation	0	0	0	0	0	0	0	0	71.80	71.22	0.58	72.51	−0.71
73	Training	0	0	0	0	0	0	0	0	74.11	71.22	2.89	72.51	1.60
74	Validation	0	0	0	0	0	0	0	0	74.33	71.22	3.11	72.51	1.82
75	Validation	0	0	0	0	0	0	0	0	71.54	71.22	0.32	72.51	−0.97
Actual value of the independent variable										X1, pH; X2, incubation time (h); X3, initial CoSO_4_⋅7H_2_O (ppm); X4, glucose (%); X5, glycerol (%); X6, peptone (%); X7, K_2_HPO_4_ (%); X8, MgSO_4_.7H_2_O (%).				
Low (−1)		5.5	24	100	0.5	0.5	1.5	0.10	0.10					
Center (0)		7.0	48	150	1.0	1.0	2.0	0.15	0.15					
High (1)		8.5	72	200	1.5	1.5	2.5	0.20	0.20					

Before the biosorption trials, the bacterium inoculum was prepared freshly from 48 h aged culture after growing on the broth medium of Atlas and Snyder (2006) under shaking at 100 rpm and 28 ± 1°C for 48 h. The bacterial count was adjusted to 10^8^ cfu ml^–1^, using a hemocytometer.

### Biosorption Procedure by Definitive Screening Design

The liquid-state biosorption technique was applied for constructing the DSD, assuming the lake of differences among all studied criteria. The relative importance and significance of medium components were investigated to manage the cobalt bioleaching process. For DSD construction, a total of 8 continuous independent variables of biosorption conditions were tested at three numeric levels, namely, two corner points (low (−1), and high (+1) levels) and one center (0) level located at the midway between low and high settings. The coded levels and actual values of the matrix of the DSD are presented in Table 1. The relation between the actual and the coded values of the tested parameters was calculated using the following equation (Abou Ayana et al., 2015):

$$x_{\text{i}}=\left({\text{X}}_{\text{i}}-X_{0}\right)/\Delta \,{\text{X}}_{\text{i}}$$

where X_*i*_ is the coded value of an independent factor, Δ*X*_*i*_ is the step change in the actual value of the variable i, X_0_ is the actual value of an independent factor at the center point, and Xi is the actual value of an independent factor.

*Pseudomonas alcaliphila* inoculum (10% v/v) was used to inject 45 ml of broth medium in 250 ml Erlenmeyer flasks. Following the various combinations of design reported in the DSD matrix, the inoculated runs were incubated at 28 ± 1°C in a shaker at 100 rpm. After the biosorption process, bacterial cells were separated by centrifugation (5,000 rpm for 20 min). The supernatant of the various runs was examined for the residual cobalt (Xie, 2003) using inductively coupled plasma (ICP) spectrometry (model Ultima 2 JY Plasma, Horiba, France). The capacity of *P. alcaliphila* as a cobalt biosorbent was determined as follows:

$$\mathrm{Cobalt}\,\mathrm{removal}\,\mathrm{efficiency}\%=\frac{\text{C}\,1-\text{C}\,2}{\text{C}\,1}\times 100$$

where C1 and C2 are the initial (control) and residual cobalt concentrations, respectively.

### Modeling of Cobalt Removal Using Artificial Neural Network

The ANN model was fed with the data from the DSD matrix (Table 1). A platform of connected neural networks was established with a hidden layer that contained nodes with the same (i.e., NTanH) hyperbolic tangent sigmoid activation function. A multilayer perceptron algorithm that is fully connected was used to create the prediction of the ANN from the data obtained in the DSD matrix. Three data sets were created after the randomization of the data. The first was used for training (using 50 runs to minimize prediction error and establish neural weights), the second was used for validation (using 25 runs to stop ANN training and selection of the best model, with a holdback propagation of 0.3333), and the third used as an external data set to test for the robustness of the ANN model, i.e., the final assessment of prediction capabilities, which was then used in the final evaluation and excluded from model selection. The design ANN topology is composed of the input layer represented by the eight independent factors, and the output layer that has one neuron (cobalt bioremoval by *P. alcaliphila* NEWG-2) in which both had a fixed number of independent and response factors that were tested, respectively. Between the two layers, another hidden layer(s) was constructed and tested; this in-between layer(s) was examined using several neurons (ranging from 3 to 10) at various learning rates. The ANN is designated as 8-h-1.

Machine learning continued until obtaining the minimum values of root mean square error (RMSE), mean absolute deviation (MAD), and the sum of squared errors (SSE), with the highest value of the coefficient of determination (R^2^), as well as the predicted outputs closely matching the actual effect of Co(II) biosorption. The fitness of the models generated by the DSD and ANN was compared with the corresponding experimental values.

### Scanning Electron Microscopy

To evaluate the removal levels of Co^2+^ inside cells, scanning electron microscopy (SEM) inspection was carried out by coating the surface of the *P. alcaliphila* NEWG-2 with gold. Broth medium containing the ANN-optimized conditions of 8.5 pH, 67.5 h of incubation time, 200 ppm of initial concentration of CoSO_4_⋅7H_2_O, 1.5% glucose, 1.5% glycerol, 1.5% peptone, 0.2% K_2_HPO_4_, and 0.2% MgSO_4_.7H_2_O was used. Bacterial cells were separated by centrifugation at 5,000 rpm for 20 min, coated with gold, and examined at various magnifications using SEM (JEOL TEM-2100) attached to a charge-coupled device (CCD) camera under an accelerating voltage of 200 kV at Central Laboratory, Electron Microscope Unit, Mansoura University, Egypt.

### Energy-Dispersive X-Ray Analysis

Energy-dispersive X-ray analysis was performed through SEM; a JEOL TEM-2100 connected to a CCD camera at an accelerating voltage of 30 kV was used to conduct an EDX analysis at the Central Laboratory, Electron Microscope Unit, Mansoura University, Egypt.

### Fourier Transform Infrared Spectroscopy

*Pseudomonas alcaliphila* NEWG-2 cells were prepared according to the same conditions for SEM inspection and then analyzed before and after Co(II) removal using the Fourier transform infrared spectroscopy (FTIR) spectroscopy and KBr pellets to determine the active groups. The FTIR spectra of *P. alcaliphila* NEWG-2 were predicted in the range, being 400 to 4,000 cm^–1^ using Thermo Fisher Nicolet IS10, United States spectrophotometer, at Spectral Analyses Unit, Mansoura University, Egypt.

### Trial Design and Statistical Checkup

The package of JMP pro software, version 16.2 (JMP^®^, SAS Institute Inc., Cary, NC, United States), was used for the construction of the DSD matrix and data analysis, as well as, for establishing the ANN topology. By utilizing machine learning with hidden neurons that train, validate, and test the experimental data, 50 randomly selected runs were used for the training of the ANN and 25 separate random runs were used for checking up on the validity of the ANN model training and to boost the accuracy of the ANN prediction. To boost the forecast accuracy of the models, DSD experiments were repeated three times.

## Results

### Exploring the Medium Criteria Using the Definitive Screening Design Paradigm

Eight independent factors as reminded in the DSD matrix were investigated for their influence on the biosorption of Co(II) by *P. alcaliphila* NEWG-2. The experiments were performed, following the matrix of the screening design, DSD (Table 1). The paradigm of the design was based on the null hypothesis (H_0_) that assumed an equal effect of the tested independent factors and without correlation between the variables and biosorption of Co(II). The experimental results reveal apparent variation among the 75 runs of cobalt biosorption percentages.

The values obtained experimentally from several DSD combinations were statistically checked to determine the factors that significantly affect Co(II) biosorption. The predicted value percentage of Co(II) sorption was comparable with the experimental ones. Moreover, the residual values from the differences between experimental and predicted values were also small.

**FIGURE 1:**
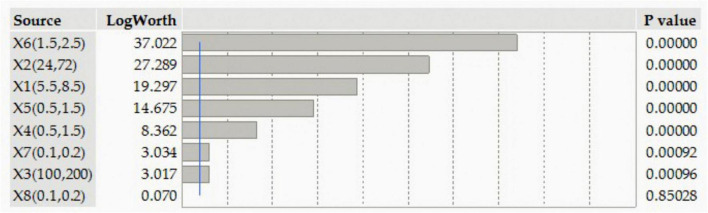
The relative importance of each of the tested independent variables on the bioremoval of cobalt by *P. alcaliphila.* X1, pH; X2, incubation time (h); X3, initial CoSO_4_.7H_2_O (ppm); X4, glucose (%); X5, glycerol (%); X6, peptone (%); X7, K_2_HPO_4_ (%); X8, MgSO_4_.7H_2_O (%).

Figure 1 is generated to determine and interpret the contribution of the independent variable(s) to the variability of Co(II) sorption. The LogWorth value and *P*-value of the tried factors are figured in the descending order. All the tested factors surpassed the edge of significance level (*P* < 0.05), except MgSO_4._7H_2_O. Among the significant factors, peptone had a superiority, whereas the initial cobalt concentration was inferior.

For extra evaluation of the null hypothesis, the coefficients and analysis of variance (ANOVA) of Co(II) sorption data were estimated (Table 2). The values of the regression coefficients for each of the tested parameters were calculated and found to vary from positive to negative values. The threshold of the significant influence of the tested variables was set at *P* < 0.05, meaning that variable(s) having P less than 0.05 is considered significant.

**TABLE 2:** Regression coefficient (coded units) and analysis of variance of the DSD experimental Co (II) bioremoval data by *P. alcaliphila* NEWG-2.

Source		Coefficient	Freedom degree	Sum of squares	Mean square	F ratio	Prob > F*
Model		71.218	8	9535.00	1191.88	180.64	< .0001
Error	Lack-of-Fit	–	12	246.71	20.56	5.88	<0.0001
	Pure error	–	54	188.76	3.50	–	-
	Total	–	66	435.47	6.60	–	-
Corrected Total		–	74	9970.48	–	–	-
Linear	X1	4.579	1	1131.97	1131.97	171.56	<0.0001
	X2	6.521	1	2296.06	2296.06	347.99	<0.0001
	X3	−1.208	1	78.83	78.83	11.95	0.001
	X4	2.362	1	301.36	301.36	45.67	<0.0001
	X5	3.607	1	702.51	702.51	106.47	<0.0001
	X6	−9.569	1	4944.63	4944.63	749.41	<0.0001
	X7	−1.213	1	79.40	79.40	12.03	0.0009
	X8	0.066	1	0.24	0.24	0.04	0.8503
**The goodness-of-fit statistics of the DSD model**							
Standard deviation			12.241				
Coefficient of determination (R^2^)			0.9563				
Adjusted-R^2^			0.9510				
Predicted-R^2^			0.9424				
Akaike’s information criterion			368.199				
Bayesian information criterion			387.936				
Predicted residual error sum of squares			574.570				

X1, pH; X2, incubation time (h); X3, initial CoSO_4_.7H_2_O (ppm); X4, glucose (%); X5, glycerol (%); X6, peptone (%); X7, K_2_HPO_4_ (%); X8, MgSO_4_.7H_2_O (%). *Significant level threshold was at P < 0.05.

The ANOVA showed that the overall model term is statistically significant (*P* < 0.0001). The next procedure was, therefore, to figure out the association between cobalt bioremoval data by *P. alcaliphila* and each factor in the design by comparing with the *P*-value of each term. All tested parameters have a statistically significant association with cobalt bioremoval; MgSO_4_ was the only exception. However, as specified by the regression coefficient, MgSO_4_ had an insignificant positive effect, whereas the initial CoSO_4_ concentration, peptone (%), and K_2_HPO_4_ (%) had a significant negative impact, and the other factors had significant positive ones.

Certain statistics were estimated for further assessing the aptness of the DSD model. The standard deviation was 12.241. The values of R^2^, adjusted-R^2^, and predicted-R^2^ show high values, being 0.9563, 0.9510, and 0.9424, respectively. The values of the corrected Akaike’s information criterion (AIC), the Bayesian information criterion (BIC), and the predicted residual error sum of squares (PRESS) are relatively small, being 368.199, 387.936, and 574.570, correspondingly.

**FIGURE 2:**
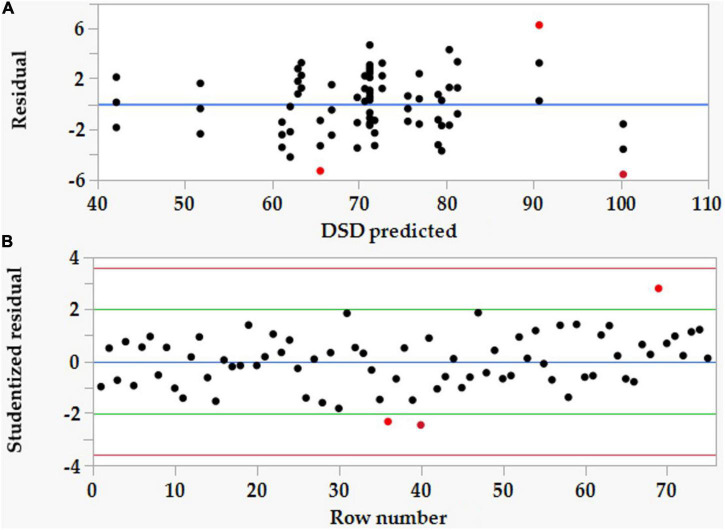
The plots of residual vs. predicted values **(A)** and the standardized residual vs. raw number **(B)** of the definitive screening design (DSD) data of Co (II) removal by *P. alcaliphila* NEWG-2.

Opposite to the above-mentioned statics, the lack-of-fit error showed to be significant (*P* < 0.0001) behavior, which did not support the model aptness. Therefore, the residuals’ analysis was checked to verify the forecasting ability of the model. The adequacy of the assumptions of the analysis of DSD data was checked by employing the residual analysis. Plotting the frequency of the residual vs. predicted (Figure 2A) values, as well as the standardized residual vs. raw number (Figure 2B), indicates that the residuals are distributed randomly but evenly along the two 0-axis sides, but there were three extreme outlier points (highlighted in red, No. 36, 40, and 69) that had high residual values. These points are considered weak points in the DSD model.

Nevertheless, the (H_0_) was rejected, and the alternative hypothesis (H_1_) was accepted since seven of the eight parameters showed a significant effect; hence, data were used for model generation. Accordingly, the regression equation in coded units is generated as follows:

Cobaltbioremoval(%)=71.218+4.579⁢(X⁢1)+19.17⁢(X⁢2)-1.208⁢(X⁢3)+2.362(X4)+3.607⁢(X5)-9.569⁢(X6)-1.213⁢(X7)+0.066⁢(X8)

where X1; pH, X2; incubation time (h), X3; initial CoSO_4_⋅7H_2_O (ppm), X4; glucose (%), X5; glycerol (%), X6; peptone (%), X7; K_2_HPO_4_ (%), and X8; MgSO_4_.7H_2_O (%).

Depending upon the data analysis of the DSD matrix, the operating conditions of bioremoval of cobalt involve seven significant independent variables, whereas MgSO_4_.7H_2_O was insignificant. Unfortunately, the performance of the DSD model was invalid for forecasting the prediction of cobalt removal, where the behavior of the lack of fit showed to be significant, whereby the previous equation could not be valid for the prediction of cobalt removal efficiently. Consequently, the design matrix of DSD and its data were further modeled using the ANN paradigm.

### Modeling Cobalt Bioremoval by Artificial Neural Network

The responded data of the DSD design (Table 1) was used for machine learning and emerging the predictive ANN model, constructing a multilayer feed-forward fully connected neural network ANN architecture platform to model bioremoval of cobalt by *P. alcaliphila*. Numerous hidden layers and neurons were utilized to determine the best architectural structure by testing various combinations of ANN-specific parameters such as the learning rate with each node sharing the same NTanH. Machine learning was used to validate the constructed ANN using the holdback procedure at a proportion of 0.3333. The trial and error continued until the maximal R^2^ was reached.

**FIGURE 3:**
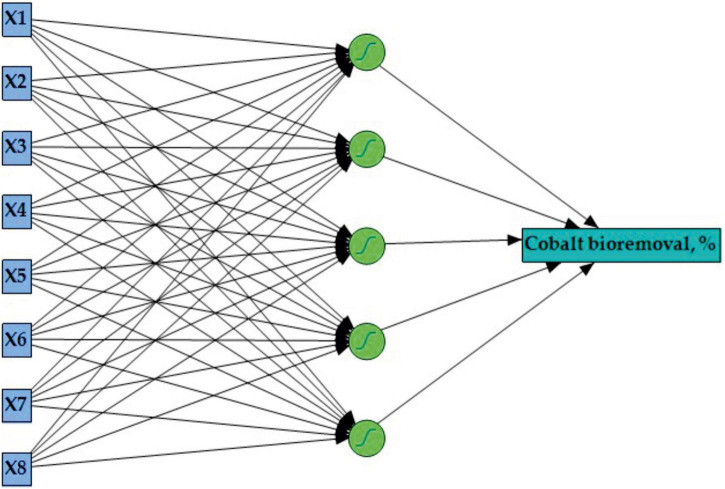
The general layout of the proposed artificial neural network for cobalt bioremoval by *P. alcaliphila* shows an input layer with eight neurons, a hidden layer with five neurons, and an output layer with one neuron. X1, pH; X2, incubation time (h); X3, initial CoSO_4_.7H_2_O (ppm); X4, glucose (%); X5, glycerol (%); X6, peptone (%); X7, K_2_HPO_4_ (%); and X8, MgSO_4_.7H_2_O (%).

The best ANN combination was generated (one hidden layer with 5 neurons) using several trials of 100 tours at a learning rate of 0.1, using the method of squared penalty. The ANN topology (Figure 3) was assigned as 8-5-1 with an input layer comprising of 8 neurons for each tested independent factor and a single output layer for cobalt bioremoval. The optimal performance for the hidden layer was using 5 neurons, NTanH(5). These conditions are accompanied by the ANN’s ability to predict outputs that could be comparable with the experimental response value.

Definitive screening design and ANN predicted values (at each experimental point) were calculated and are shown in Table 1. The ANN-predicted values were much closer to the experimental values when compared with the DSD-predicted values.

### Comparison of Definitive Screening Design and Artificial Neural Network Models

**FIGURE 4:**
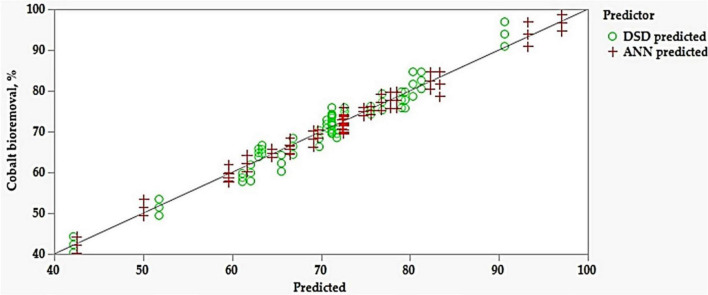
Actual by the predicted plot for cobalt bioremoval by *P. alcaliphila*.

Definitive screening design and ANN models’ performance was compared based on their accuracy in predicting cobalt bioremoval. The accuracy of training and validation of both models were measured and compared based on statistical parameters (Table 3). The ANN model had higher R^2^ values for training and validation compared with the DSD model, while RMSE and MAD recorded lower values. This trend was consistent across the dataset. The predicted values of both DSD and ANN models were plotted against the experimental values. ANN showed a higher predictive capacity than DSD (Figure 4).

**TABLE 3:** Comparison statistics of the model operation established by DSD and ANN for Co (II) bioremoval by *P. alcaliphila.*

Training statistics				
Model	R^2^	RMSE	MAD	Frequency
DSD	0.9528	2.5729	2.1433	50
ANN	0.9779	1.7607	1.4640	50
**Validation statistics**				
DSD	0.9617	2.0444	1.6868	25
ANN	0.9773	1.5728	1.2543	25

**Overall model performance**				

**Statistics**		**DSD**	**ANN**	**Frequency**

R^2^		0.9563	0.9783	75
RMSE		2.4096	1.7004	75
MAD		1.9911	1.3941	75
SSE		435.262	216.759	75

*RMSE, root mean squared error; MAD, mean absolute deviation; SSE, the sum of squares error.*

### Experimental Validation of Both Models

**FIGURE 5:**
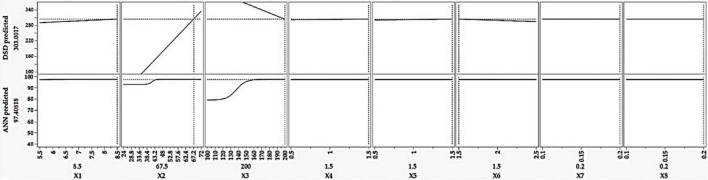
The estimated values of the tested parameters based on DSD and artificial neural network (ANN) models and the corresponding predicted cobalt bioremoval by *P. alcaliphila*. X1, pH; X2, incubation time (h); X3, initial CoSO_4_.7H_2_O (ppm); X4, glucose (%); X5, glycerol (%); X6, peptone (%); X7, K_2_HPO_4_ (%); and X8, MgSO_4_.7H_2_O (%).

The optimal mixture of the tested variables and the corresponding maximum response of cobalt bioremoval by *P. alcaliphila* were determined. The tested variables were estimated to be 8.5 pH, 67.5 h of incubation time, 200 ppm of initial CoSO_4_⋅7H_2_O, 1.5% glucose, 1.5% glycerol, 1.5% peptone, 0.2% K_2_HPO_4_, and 0.2% MgSO_4_.7H_2_O. These estimates were evaluated under laboratory conditions to check the forecast capacity of both models. The theoretical cobalt removal % by the DSD model displayed an unusual value (303.0%). The ANN, in contrast, achieved an effective and meaningful predicted removal percentage of cobalt, being 97.41%. These values were validated under laboratory conditions, and the experimental value obtained was 96.32 ± 2.1%, which was in line with estimated values by the ANN model. However, Figure 5 shows the array of every single factor while keeping the other seven factors constant.

### Surface Morphology Analysis

**FIGURE 6:**
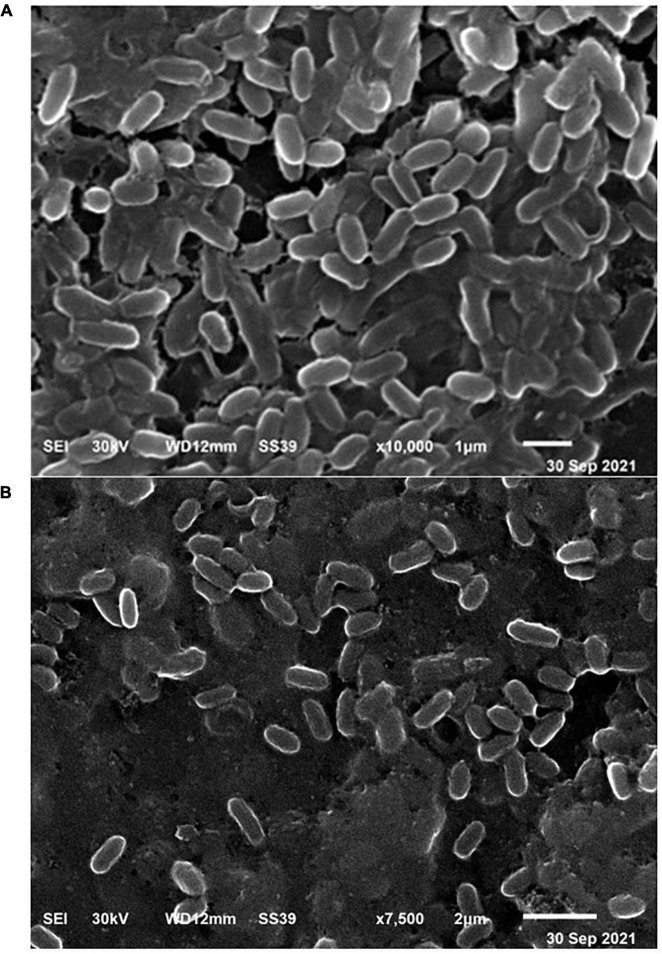
Micrograph of scanning electron microscopy, viewing the normal cells of *P. alcaliphila* NEWG-2 **(A)** before and **(B)** after biosorption process of Co (II).

To summarize, the acclimatization of bacteria by Co(II) ions as shown in Figures 6A,B, was investigated *via* the photography of the bacterial surface by SEM, which indicated that the irregular surface of *P. alcaliphila* NEWG-2, with swelling of the cells, appeared after adsorption of Co(II) ions, whereas the normal surface of bacterial cells was of regular shape, before Co(II) ions adsorption. This illustrates the surface morphology changes and effects of Co(II) on the bacterial cells.

### EDX Evaluation

**FIGURE 7:**
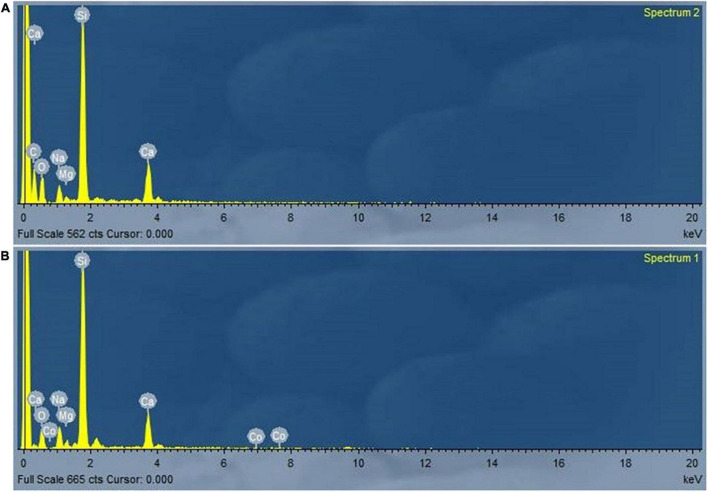
Analysis of electron dispersive spectroscopy of *P*. *alcaliphila* NEWG-2 presents the normal cell element before **(A)** treatment in comparison with the emerging peak of Co (II) ions after **(B)** the biosorption process.

The EDX analysis was conducted to determine the insertion of Co(II) into the cell wall of *P. alcaliphila* NEWG-2 as the biosorption of Co(II) ions; the data are described in Figures 7A,B). Depicted data as shown in Figure 7 indicated the presence of extra peaks after the biosorption course.

### Fourier Transform Infrared Spectroscopy Examination

**FIGURE 8:**
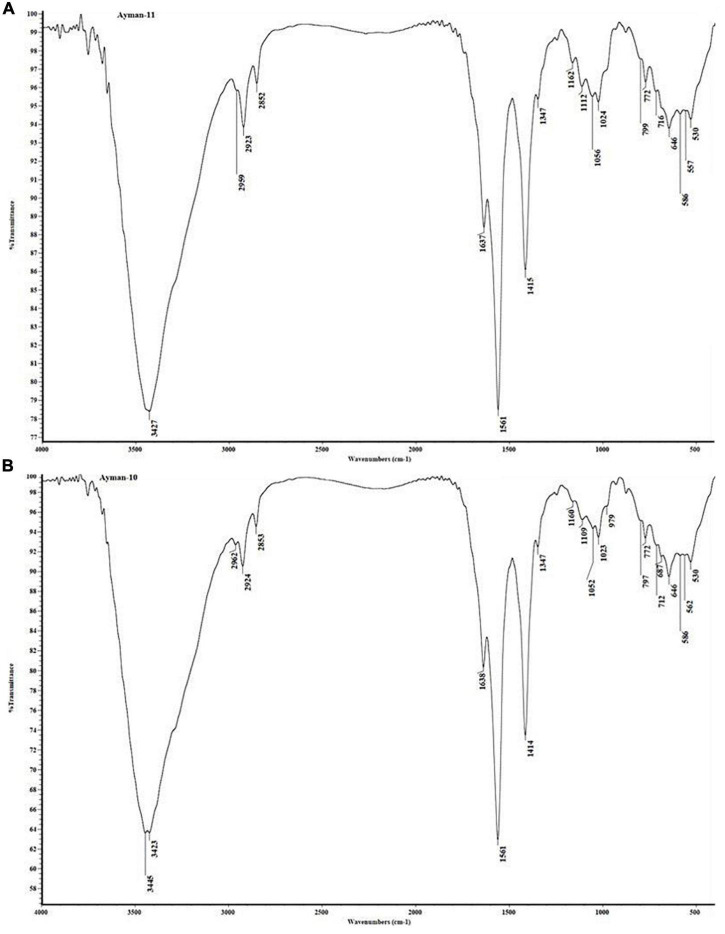
Variation in bands between the untreated **(A)** and treated **(B)** cells as a result of Co (II) ions, as detected by the Fourier transform infrared spectroscopy analysis of *P*. *alcaliphila* NEWG-2.

The FTIR spectra of the dry biomass of *P. alcaliphila* NEWG-2 were monitored before and after Co (II) biosorption. The variation in the content could be due to the attachment of Co(II) with functional groups of the cell wall of *P. alcaliphila* NEWG-2. The results referred to the characteristic frequencies, and the interpreted functional groups are publicized in Figure 8 and Table 4. The interaction of Co (II) ions with the *P. alcaliphila* NEWG-2 surface led to a change in the morphological features and surface properties. Generally, the infrared (IR) spectral analyses were employed to scrutinize the characteristic functional groups of *P. alcaliphila* NEWG-2 and investigate the changes associated with the frequencies of the functional groups before and after biosorption of Co (II) ions as a result of forming bonds between them, which led to the creation of new bonds or the diffraction of some group frequencies. Whereby, the IR spectra of two samples, e.g., *P. alcaliphila* NEWG (control) and *P. alcaliphila* NEWG-2 treated with cobalt ions, were investigated. The magnitude of the recorded frequencies analysis was expressed as the wavenumber in the range of 400–4,000 cm^–1^.

**TABLE 4:** Fourier transform infrared spectroscopy (FTIR) spectral analysis for *P*. *alcaliphila* NEWG-2 before and after biosorption of Co (II) ions.

*Pseudomonas alcaliphila* (Control)		*Pseudomonas alcaliphila* + Cobalt	
Wave no. (cm^–1^)	Functional groups	Wave no. (cm^–1^)	Functional groups
3,427	Strong, broad O-H stretching	3,445, 3,423	Strong, broad O-H stretching
2,959	Strong, broad N-H stretching	2,962	Strong, broad N-H stretching
2,923	Medium C-H stretching	2,924	Medium C-H stretching
2,852	Medium C-H stretching, aldehyde	2,853	Medium C-H stretching, aldehyde
1,637	Strong C = O stretching, amide	1,638	Strong C = O stretching, amide
1,561	Medium C = C stretching	1,561	Medium C = C stretching
1,415	Strong S = O stretching	1,414	Strong S = O stretching
1,347	Medium C-H bending	1,347	Medium C-H bending
1,162	Strong C-O stretching	1,160	Strong C-O stretching
1,112	Strong C-O stretching, secondary alcohol	1,109	Strong C-O stretching, secondary alcohol
1,056	Strong C-O stretching, primary alcohol	1,052	Strong C-O stretching, primary alcohol
1,024	Strong S = O or Si-O-Si stretching	1,023	Strong S = O or Si-O-Si stretching
		979	Strong C-H bending, 1,2-disubstituted
799, 772	Strong C-H bending, 1,2,3-trisubstituted	797	Strong C-H bending, 1,2,3-trisubstituted
716	Strong C-H bending, monosubstituted	772, 712	Strong C-H bending, monosubstituted
		687	Symmetric NH_2_ bending
646, 586	Strong C-X stretching or aromatic ring	646, 586	Strong C-X stretching or aromatic ring
530	Strong L-X stretching	562, 530	Strong L-Co stretching

Data of the IR spectral analyses prohibited that the absorption band at ν = 3,423–3,445 cm^–1^ is attributed to the strong or broad stretching vibration of hydroxyl “O-H” groups. The absorption bands in the range of ν = 2,923–2,960 cm^–1^ are assigned to the vibrations of a strong, broad N-H stretching group. The absorption bands at ν = 2,923–2,925 cm^–1^ are assigned for medium stretching C-H groups with a slight shift in values (+1 and +2) of the *P. alcaliphila* NEWG-2 treated with cobalt ions. The absorption bands at ν = 2,852–2,856 cm^–1^ are identified for medium stretching C-H of aldehyde groups for the two samples. Interestingly, a new absorption band was recorded at ν = 2,232 cm^–1^ owing to a weak stretching C≡N group “nitrile” in the analysis of *P. alcaliphila* NEWG-2 treated with cobalt ions.

The values of the frequencies within the range ν = 1,637–1,024 cm^–1^ in the spectral IR analysis were characterized for eight groups in all samples without any appearance or disappearance of the absorption bands along with slightly shifted values. Thus, the absorption bands due to strong stretching (C = O, amide), medium C = C, strong S = O, medium C-H bending, strong C-O, strong C-O stretching “secondary alcohol”, strong C-O stretching “primary alcohol,” and strong stretching S = O or Si-O-Si groups were identified at ν = 1,634–1,638, 1,561–1,562, 1,414–1,416, 1,347–1,349, 1,160–1,162, 1,109–1,114, 1,052–1,059, and 1,023–1,024 cm^–1^, respectively.

The results of the IR spectral analysis of *P. alcaliphila* NEWG-2 demonstrated no absorption bands appeared in the range of ν = 935–980 cm^–1^, and the IR spectra of the other samples recorded absorption bands in this range owing to strong C-H bending groups. It also found that the values are recorded at ν = 772–880 cm^–1^ that is attributed to strong C-H bending; “1,2,3-trisubstituted” did not include any appearance of the disappearance of the absorption bands, but these values were slightly shifted than their value in the IR spectrum of the control sample. No absorption band was recorded in the range of ν = 683–687 cm^–1^ in the analysis of the control sample, while new absorption bands were recorded in the treated samples with cobalt ions owing to the absorption bands of symmetric amino (NH_2_) binding. The values of frequencies at ν = 582–586 and 624–646 cm^–1^ are attributed to the stretching vibration bands of C-halogen groups or aromatic rings. Furthermore, the IR spectra of *P. alcaliphila* NEWG-2 (control) indicated an absorption band at ν = 530 cm^–1^ due to a strong stretching vibration of halogen linked to the ligand, while the values at ν = 562, 530, and 531 cm^–1^ are attributed to the strong stretching vibration of the new bond formed with cobalt ions.

## Discussion

Environmental pollution could be correlated with an improper discharge of urban and industrial effluents, in addition to a lack of sanitation procedures. Industrial effluents usually contain some of the heavy metals that cause disturbance of the ecosystem creatures, such as aquatic plants, algae, and invertebrates (Stubblefield et al., 2020). However, at present, it is crucial to restore the balance of the ecosystem through perspective plans, with the creation of technical procedures for the restoration of such a natural ecosystem (Abhilash, 2021). The bioremediation process is one of the favorable techniques, by which the process is performed using microorganisms, with advantages of efficiency, adequate, and having inherent capacity to remove and/or minimize these pollutants (Cajthaml, 2015).

Wherein, this investigation has been designed to attain a comparative study of the optimization process of biosorption of Co(II) by *P. alcaliphila* using DSD and ANN models.

Concerning the DSD paradigm, the optimization process of biosorption medium with eight independent variables, i.e., peptone, pH, incubation periods, thresholds of Co(II), MgSO4, K_2_HPO_4_, glucose, and glycerol, was conducted. The relative analysis of ANOVA of DSD selected seven of eight tested variables that were significant for bioremoval of Co(II). The *P*-value < 0.05 of the overall design means the DSD model is significant, and this is approved by the goodness-of-fit measurements. Additionally, it had been shown that from the DSD model, the predicted values of cobalt removal percentage were comparable with experimental ones. The R^2^, adjusted R^2^, and predicted R^2^ were in rationally high values, being 0.9563, 0.9510, and 0.9424, respectively. The standard deviation was 12.241. The residual values as the difference among experimental and predicted values showed to be comparable as well. The AIC, BIC, and PRESS statics are also the goodness-of-fit statistics with relatively small values, being 368.199, 387.936, and 574.570, respectively, which confirm the aptness of the DSD model. Moreover, among the interactions between independent variables and removal percentage of Co(II), peptone came up with a significant superiority during removing percentage, followed by an incubation period, pH, glycerol, and glucose, respectively, whereas the initial thresholds of Co(II) have significant inferiority of bioremoval percentage. Furthermore, the MgSO_4_ showed to have a negative impact on biosorption removal of Co(II).

Peptone is the proteinaceous compound that is necessary for bacterial growth since it is required for RNA synthesis, shifting bacterial cells from lag to exponential phase, source of amino acids for energy transduction, and repairing the DNA damage during oxidative stress (Rolfe et al., 2012). Furthermore, there is a correlation between intracellular protein satisfaction and biosorption of heavy metals, indicating some protein fragments are key molecules to binding heavy metals (Bhatia et al., 2011), as well as, the biosorption of the heavy metal was influenced by the type of amino acids, i.e., basic and acidic amino acids (Kim et al., 2007).

The KH_2_PO_4_ had a significant effect as the main source of the essential inorganic phosphate mineral, which is a common component of nucleotides, membranes, and phospholipids, as well as several phosphorylation events within the bacterial cell (Clements et al., 2002). The transmission of phosphate in most species of bacteria is due to induced transcription of the phosphate uptake genes (Bruna et al., 2022).

Otherwise, MgSO_4_ salt had a negative impact on the biosorption process. Although, the metals play a crucial role as a fundamental for central metabolic processes (Hobman et al., 2005). Metal salts are necessary for homeostasis and the prevention of metal toxicity in the cell (Giedroc and Arunkumar, 2007). Magnesium and sodium were found to be at maximal concentrations in the mid-exponential phase of bacteria; the MgtA genes are discovered in the lag phase, which could be responsible for increasing magnesium during the exponential phase (Rolfe et al., 2012).

The uptake of metals, i.e., Co, Cu, Pb, Ni, Mn, Zn, and Cr was influenced by incubation temperature, pH, and incubation time (Kim et al., 2007). The investigated biosorption of Co(II) ions by *P. aeruginosa*, as influenced by pH values, incubation periods, and thresholds of Co(II), has been investigated (Dharanguttikar, 2018). The biosorption process could be dependent upon ion exchange, where the sodium ion was found to take part in the uptake of cobalt into *P. aeruginosa* (Dharanguttikar, 2018). The pH value has a crucial role during the Co(II) biosorption, in which the upregulating pH can increase the adsorption of Co(II). Contrarily, low pH causes low adsorption due to competition between metal cations and protons to the active site. As well, the electrostatic repulsion between positively charged surface sites and positive metal cations causes the reduction of adsorption of cobalt (Khajavian et al., 2020). The efficiency of the biosorption process of lead, cobalt, and chromium could be due to the alginate content of *Pseudomonas* spp. and culture conditions (Conti et al., 1994; Khraisheh et al., 2020; Meena et al., 2020).

Both glucose and glycerol are crucial for energy and fatty acids for the growth of microorganisms, with the shortage of glucose or other sugars the organism obligately searching alternative ones, such as protein or fat, for using it as a source of carbon, so the occurrence of glucose or other sugars is necessary for metabolic process, e.g., glycolysis cycle.

Meena et al. (2020) investigated the productivity of alginic acid (exopolysaccharide), a factor chelating metals of *Pseudomonas stutzeri* during the medium containing sucrose with other components. Although the fitness of the aforementioned parameters of the analysis, there are exceptional defects apparently from depicting the residual vs. the DSD predicted, as well as, the standardized residual vs. run number values, which showed unusual points scattered away from the centerline (0-axis), including unidentified values, causing the poorness of the predictive ability of the DSD model.

Additionally, the lack-of-fit error was shown with a significant value. The theoretical value of the Co(II) removal percentage was peculiar, being 303.0%; consequently, the model aptness could not be enhanced. Furthermore, this model was assumed to be invalid, with improbability to deduce the predicted equation. However, this model showed potentiality in distinguishing significant factors. Other reported studies indicated the availability of the DSD model for determination of the significant factor, estimating the main effect, and studying the interaction between the two factors (Jones and Nachtsheim, 2011; Lin, 2015).

Regarding the ANN paradigm, its construction is depending upon feedback from the dataset of DSD; among the data analysis by ANOVA, the coefficient of determination (R^2^) was shown at a reasonable value (0.9779). The lack of fit was significant.

The data analysis also showed that the value between the actual and predicted ANN is close, which signifies the nonlinear fitting effects of the good model. Additionally, the values of RSME, MAD, and SSE are small compared with those of the DSD model. Besides, the reasonable value of R^2^ and the lower value of RSME of the ANN model confirmed the accuracy of the model (Gurney, 2018). The predicted value of Co^2+^ removal was found in the appreciable value of 97.41%. Whereby, the ANN is valid with the capability to predict the equation prediction, due to extended predictive capacity with accuracy in studying the nonlinearity system (Ram Talib et al., 2019; Saber et al., 2021).

However, the ANN model could not deduce the relation between inputted and outputted factors, so the model utilizes the overall factors under study (Shafi et al., 2018; Saber et al., 2021).

Eventually, our data indicated that the DSD was not credited comparatively with ANN, in addition to the availability of ANN for studying the optimization for such as these biosorption processes. However, both DSD and ANN have success in modeling biomolecules, i.e., citric acid productivity by *Trichoderma* sp. (Elsayed et al., 2021). Commonly, in comparison with the DSD model, the ANN paradigm has a more generalization capacity; this could be due to its universal ability to approximate the nonlinearity of the system. Additionally, the ANN paradigm can inherently capture almost any form of nonlinearity, since it effortlessly overcomes the above-conferred restriction of DSD.

Whereby, in the case of ANN, the generous search space could be preferred, even if the correlation in that space is more convoluted than quadratic (Desai et al., 2008). Overall, the ANN model includes the choice of architecture network, hidden layer determination, number of neurons, learning, training, validation, and verification of the data (Gurney, 2018). Other studies pointed out the consistency of the ANN model for optimized leaching of Cr ions (Ram Talib et al., 2019; Saber et al., 2021). The exhausting ANN model has been successfully applied in the biosorption of some heavy metal ions (Geyikçi et al., 2012; Ghosh and Sinha, 2015).

Concerning surface characteristics of *P. alcaliphila* NEWG-2, the photography as shown by SEM illustrated the irregular cell surface after absorption of Co(II) ions, with an enlarged and swelling size of the cell, while the surface of cells showed to be regular, with the normal size before Co(II) treatment. This phenomenon of the morphological characteristics was in harmony with the investigation of Kim et al. (2007), who pointed out that heavy metals were distributed among cell wall and cell membrane fractions, causing alterations in the cell morphology. However, it is worthy to mention that the morphology is not significantly changed, that observation can be explained based on the ability of the current bacterium to absorb and tolerate high concentration of Co(II) ions, thus leading to or resulting in the bacterial ability to stand against significant alteration in the morphology.

Furthermore, the EDX analysis is generally used to explore the elemental content of the biosorbent, such as Co(II) ion during the biosorption process by bacterial cells (Dmytryk et al., 2014). The depicted data indicated the additional column of Co(II) ions appeared during the biosorption process by *P. alcaliphila* NEWG-2. Similarly, another study reported the capability of *P. alcaliphila* in the biosorption of Cr^6+^ during the EDX analysis (Saber et al., 2021). Moreover, the FTIR analysis of dried cells of *P. alcaliphila* was determined, and the analysis has been carried out at the wavenumber range of 400–4,000 cm^–1^. Commonly, the biosorption process has been associated with a change in morphological and cell surface features, in addition to the change in frequencies of functional groups, with the creation of new bonds. The functional groups, i.e., nitrile, hydroxyl, amino, S-O, and aldehyde groups, were detected during the analysis of dried cells of *P. alcaliphila* NEWG-2. Similarly, the previous study by Dharanguttikar (2018) investigated the functional group that binds with cobalt in *P. aeruginosa*; the amine, hydroxyl, carboxyl, and phosphate esters play a crucial role in the binding of cobalt. Additionally, the functional groups, e.g., hydroxyl, carboxylate, phosphate, and amino groups, were active in binding some of the metal ions (Javanbakht et al., 2014).

## Conclusion

In this work, the optimization of Co(II) biosorption using *P. alcaliphila* NEWG-2 was conducted during the two consecutive paradigms, namely, DSD and ANN. Throughout the DSD model, peptone has superiority over other ones of biosorption medium. Along with ANOVA analysis of the DSD paradigm, the R^2^ was in reasonable values, but the lack-of-fit error showed significant behavior, so the DSD showed to be invalid and could not be applied for the forecasting of the predicted equation of Co(II) removal. Contrarily, the ANN paradigm showed high prediction ability with lower values of errors (i.e., RMSE, MAD, and SSE) compared to the DSD model. Consequently, the ANN model was comparatively valid for the optimization of the bioremoval process. The SEM and EDX investigations confirmed the ability of *P. alcaliphila* NEWG-2 in biosorption of Co(II). The functional groups of *P. alcaliphila* NEWG-2 showed to be hydroxyl, nitrile, amino, S-O, and aldehyde groups. Depending upon this study, and with aid of artificial intelligence, *P. alcaliphila* NEWG-2 exhibits strong potentiality as a candidate for the biosorption of Co(II) ions from wastewater and the environment.

Finally, the optimum estimated levels of the tested variables by ANN were 8.5 pH, 67.5 h of incubation time, 200 ppm of initial concentrations of CoSO_4_⋅7H_2_O, 1.5% glucose, 1.5% glycerol, 1.5% peptone, 0.2% K_2_HPO_4_, and 0.2% MgSO_4_.7H_2_O. These levels were checked under laboratory conditions, and the experimental bioremoval of cobalt was maximized to be 96.32 ± 2.1%. So far, this is the first attempt to optimize the biosorption of Co(II) by *P. alcaliphila via* DSD and artificial intelligence paradigms.

## Data Availability Statement

The original contributions presented in the study are included in the article/supplementary material, further inquiries can be directed to the corresponding author/s.

## Author Contributions

AE, ZM, AG, AE-K, and WS contributed to the conception and design of the study. MA, SA, AE-K, and WS organized the database. ZM, AE, and WS performed the statistical analysis. AG, AE, ZM, MA, SA, and WS wrote the first draft of the manuscript. ZM, AE, AG, and WS wrote the discussion section. AE, ZM, AG, and WS substantially contributed to the conception of the work and interpretation of data and critically revised the intellectual content of the manuscript. All authors contributed to manuscript revision, read, and approved the final version.

## Conflict of Interest

The authors declare that the research was conducted in the absence of any commercial or financial relationships that could be construed as a potential conflict of interest.

## Publisher’s Note

All claims expressed in this article are solely those of the authors and do not necessarily represent those of their affiliated organizations, or those of the publisher, the editors and the reviewers. Any product that may be evaluated in this article, or claim that may be made by its manufacturer, is not guaranteed or endorsed by the publisher.
